# Conference to Consider State or Rate Hospital Question

**Published:** 1920-10-16

**Authors:** 


### THE FEDERATION OF MEDICAL AND ALLIED SERVICES.

### Conference to Consider State or Rate Hospital Question.

On Friday, November 5, a conference organised by the Federation of Medical and Allied Services will be called to consider the position of hospitals under the Miscellaneous Provisions Bill of the Ministry of Health. Invitations have been sent to the Royal Colleges of Physicians and Surgeons, the British Medical Association, and other bodies, with a view to collecting professional opinions. The questions asked by the Federation are: (1) Is a separate Bill for health legislation preferable to the inclusion of the subject in the Bill before Parliament? (2) Should doctors, dentists, pharmacists, nurses, etc., have representation in connection with local health authorities, local hospitals, and Poor-Law institutions? (3) Should the rates be applied to the maintenance of voluntary and other hospitals? (4) If the answer to the last question is in the affirmative, should the contributions be in respect of all classes, those below a fixed limit of income, or of certain defined diseases? A deputation to the Minister of Health is also being arranged at an early date to ask him "to withhold his approval of schemes proposed with regard to the municipal control of hospitals until the medical and allied professions have had an opportunity of expressing their opinions on the principles involved in those schemes." The Federation understands that municipal hospitals are already in process of formation, and that the approval of the Minister of Health is being sought.

